# Near-Threshold and Resonance Effects in Rotationally Inelastic Scattering of D_2_O with *Normal*-H_2_

**DOI:** 10.3390/molecules27217535

**Published:** 2022-11-03

**Authors:** Astrid Bergeat, Alexandre Faure, Laurent Wiesenfeld, Chloé Miossec, Sébastien B. Morales, Christian Naulin

**Affiliations:** 1 Univ. Bordeaux, CNRS, Bordeaux INP, ISM, UMR 5255, F-33400 Talence, France; 2 Univ. Grenoble Alpes, CNRS, IPAG, F-38000 Grenoble, France; 3 Laboratoire Aimé-Cotton, CNRS, Université Paris-Saclay, Bât 505, F-91405 Orsay, France

**Keywords:** inelastic collisions, cross-sections, water, hydrogen

## Abstract

We present a combined experimental and theoretical study on the rotationally inelastic scattering of heavy water, D_2_O, with *normal*-H_2_. Crossed-molecular beam measurements are performed in the collision energy range between 10 and 100 cm^−1^, corresponding to the near-threshold regime in which scattering resonances are most pronounced. State-to-state excitation cross-sections are obtained by probing three low-lying rotational levels of D_2_O using the REMPI technique. These measurements are complemented by quantum close-coupling scattering calculations based on a high-accuracy D_2_O–H_2_ interaction potential. The agreement between experiment and theory is within the experimental error bars at 95% confidence intervals, leading to a relative difference of less than 7%: the near-threshold rise and the overall shape of the cross-sections, including small undulations due to resonances, are nicely reproduced by the calculations. Isotopic effects (D_2_O versus H_2_O) are also discussed by comparing the shape and magnitude of the respective cross-sections.

## 1. Introduction

Water is the third most abundant molecule in the interstellar medium (ISM) and has ubiquitously been observed by ground- and space-based telescopes since its first detection in 1969 in the Orion nebula [1,2]. Water thus is a key molecule for understanding the energy balance and the physical–chemical processes that occur in these environments. Despite the low elemental deuterium abundance in the Galaxy, D/H∼10^−5^, a spectacular deuterium enrichment of various interstellar molecules, has been observed in star forming regions. Among these molecules, the deuterated water isotopologues HDO and D_2_O are of special importance because they can help to understand the origin of water in protostars [3].

D_2_O was identified for the first time in 2007 toward IRAS 16293-2422 [4] in its *para* form and detected in its *ortho* form in 2010 in the same cold and diluted protostar envelope [5], which has then allowed to derive the *ortho*-to-*para* ratio of D_2_O (< 2.6. For comparison, the thermal equilibrium value is 2).

However, in order to interpret the astrophysical observations [2] in terms of relative abundances or even local physical conditions, radiative transfer modeling is necessary, as the molecular populations are generally far from local thermodynamical equilibrium. At low density, the heavy water population is indeed determined by a competition between the radiative and collisional processes, thus requiring the knowledge of rates for collisional (de)excitation. Its principal collision partner obviously is H_2_ because of its high abundance in the cold ISM.

Inelastic collisions between molecules are fundamental processes in which energy is transferred between their relative translational motion and their internal degrees of freedom. Water–hydrogen collisions play an essential role in a large variety of properties or research fields (thermal conductivity, transport properties for water diluted in molecular hydrogen, or the dynamics of molecular hydrogen confined in the cages of clathrate hydrates) and allow to better understand the non-covalent interactions between molecules of great importance for energy applications, combustion chemistry, and astrochemistry. The efficiency of the inelastic process (i.e., whether a collision does or does not promote the molecule in a given state into another state) is governed by the state-to-state integral cross-section (ICS, *σ*), which is a function of molecular energy state, colliding partner, and relative translational energies. Excitation requires that the collision energy of the impact be sufficient to reach the upper state, which at sufficiently low temperature becomes energetically impossible. It should be noticed that the rate coefficients are calculated by averaging the product *σ* × *v_r_* over a thermal distribution of relative velocities *v_r_*.

If inelastic scattering ICS is zero below the threshold energy (the energy needed to promote the molecule from its initial state to an upper final state), above this threshold, classical mechanics predicts that it rises sharply to a maximum and then decreases smoothly at higher energies [6]. However, quantum mechanics show that at low kinetic energies, the inelastic scattering ICS of simple molecular species colliding with H_2_ do not follow such a simple threshold law but are instead highly structured, particularly in the vicinity of the thresholds of the lowest molecular rotational excitations. Such behavior of the ICS characterizes the interaction potential between the colliding partners and is very sensitive to the shape of this potential energy surface (PES).

**Figure 1:**
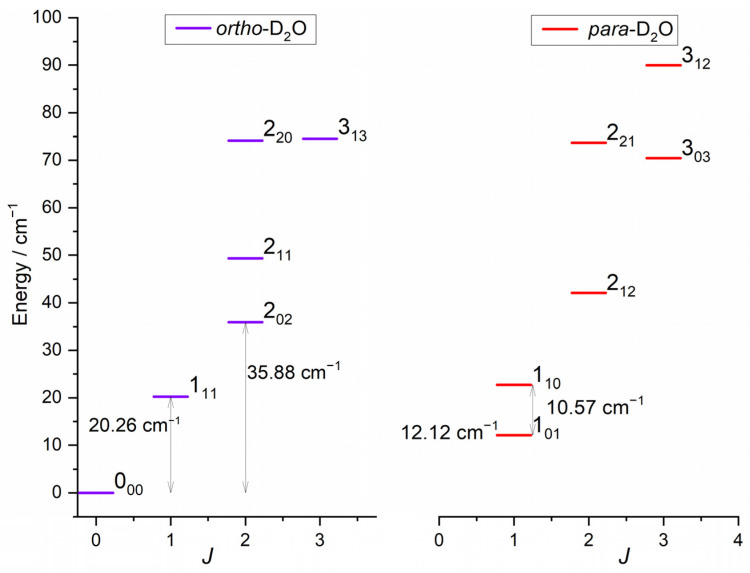
Rotational energy levels of the water isotopologue, D_2_O, for *E* < 100 cm^−1^ [7]. Levels are labelled by *j_kakc_*. Ortho levels (D_2_O, *k_a_* + *k_c_* even) are depicted in left panel and para levels in right panel.

The rotational energy levels of D_2_O are labelled by the quantum number *j_D2O_* and the two pseudo-quantum numbers *k_a_* and *k_c_*, which are the projections of *j_D2O_* along the principal inertia axis, *a*- and *c*-axes, respectively. The C_2_ symmetry axis corresponds to the *b*-axis, the *a*-axis being in-plane and perpendicular to the *b*-axis. The rotation around the *c*-axis is in the molecular plane. For a given *j_D2O_*, *k_a_* = *j_D2O_* and *k_c_* = *j_D2O_* correspond to rotation around the *a*- and *c*-axes, respectively. *Para*- and *ortho*-D_2_O levels are characterized by *k_a_* + *k_c_* odd and even, respectively, in accordance with the symmetry relations for bosons, due to the nuclear spin of D atoms. As no atom exchange is possible at the studied collision energies and as the *ortho* and *para* levels do not interconvert in inelastic collisions, these are presented separately in Figure 1. The rotational levels of H_2_ are labelled by the angular momentum *j_H2_*, with *j_H2_* even for *para*-H_2_ and *j_H2_* odd for *ortho*-H_2_ due to the fermionic nuclei.

Previous theoretical studies of the H_2_O–H_2_ interactions are too extensive to be enumerated in detail herein. The reader may refer to the last articles on rotational inelastic collisions and citations herein [8,9] and, therefore, we will limit our review to only a portion of the literature pertinent to our experiments, mainly the various experimental results on H_2_O–H_2_ and the theoretical calculations and experiments on D_2_O–H_2_.

In the first molecular beam scattering experiment on H_2_O + H_2_ elastic differential cross-sections [10], well resolved diffractive oscillations were observed, as well as the rainbow maximum. The spherically symmetric model potential parameters (e.g., the well depth and location of zero potential) were deduced assuming a Lennard–Jones (12,6) potential. The H_2_–H_2_O van der Waals complex was then studied by infrared spectroscopy in the gas phase by the group of D. Nesbitt [11,12] and also in matrix [13]. There are also studies on H_2_O pressure broadening by H_2_ [14,15] or inelastic collisions [16,17]. The Perugia group has reported determination of the potential parameters for the isotropic component of the D_2_O–D_2_ interaction by elastic scattering studies [18]. The group of Nesbitt and coworkers has also studied the infrared spectrum of the H_2_O–D_2_ complex in the bend mode frequency region [19].

A large number of studies can thus be found in the literature providing information on H_2_O–H_2_, whereas calculations or experiments on D_2_O–H_2_ are scarce. The complex has been studied in solid neon matrix [13] where the infrared absorptions principally arose from complexes involving *ortho*-H_2_, for which *j_H2_* = 1. Excitation rate constants of the D_2_O low lying rotational transitions by *para*-H_2_ were calculated for temperatures between 1 and 30 K [20] and later completed for temperatures below 100 K [21]. The calculations at the close-coupling level were performed using the highly accurate full 9D (but vibrationally averaged to 5D) PES of Valiron et al. [22]. In the cold or near cold regime (5–30 K, relevant for astrophysical applications), prominent open channel (orbiting) and closed channel (Feshbach) resonances were observed. The first state-to-state differential cross-sections (DCSs) for rotationally inelastic scattering of D_2_O(0_00_ or 1_01_) + H_2_(*j_H2_* = 0, 2 or 1) were measured by Sarma et al. [23] at the collision energy of 584 cm^−1^. At collision energies above 500 cm^−1^, this crossed-beam study could only probe the PES in the short-range and well regions. The first experimental check at long range was achieved by measuring the state-to-state scattering ICSs of rotationally inelastic excitation of D_2_O in the 2_02_ level by collisions with *para*-H_2_ [8], in the very low collision energy range 10–100 cm^1^ (near rotational thresholds). The observed scattering resonances were unambiguously identified by quantum mechanical calculations performed on the PES of Valiron et al. [22]. The aim of this study is (i) to present the full set of experimental ICSs for the D_2_O low lying rotational transitions colliding with *normal*-H_2_ in the vicinity of the thresholds and (ii) to provide new stringent tests of theory as the experimental ICS resonance structures (amplitude and position) are very sensitive to the shape and even to tiny details of the PES.

In the results section, our beam characteristics and the experimentally measured crossed beam ICSs will be presented followed by the main theoretical results in relation with our experimental studies. The experimental observations will be compared with the inelastic scattering calculations in the discussion section.

## 2. Results

### 2.1. Experimental Results

**Figure 2:**
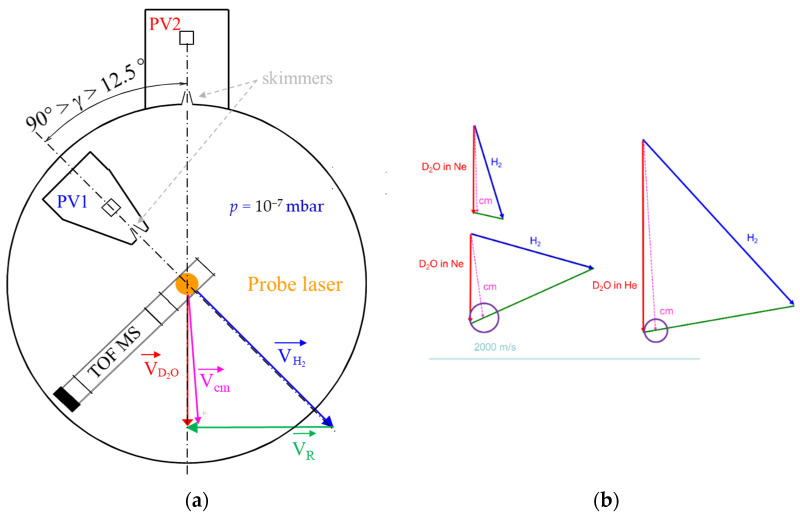
(**a**) The crossed molecular beam apparatus with variable angle, γ from 12.5° to 90°; between the two beam axes generated by pulsed valves (PV); (**b**) Newton diagrams for D_2_O seeded in Ne collisions with *normal*-H_2_ at 13° and 70° and for D_2_O seeded in He collisions with *normal*-H_2_ at 35°. The radii of the circles represent the velocities of rotationally excited D_2_O in the center-of-mass (c.m.) frame and the green vector, the relative velocities of the colliders.

The experimental results were obtained in a crossed-molecular beam (CMB) experiment under single collision conditions (see Figure 2). Our machine allows inelastic processes [24] to be studied at low collision energies. For this purpose, two supersonic molecular beams, one composed of deuterated water seeded in a carrier gas and the other of pure hydrogen, are generated with a high velocity resolution, while most of the initial population of molecular species being in their ground rotational state. The collision energy of the colliding partners is tuned by varying the beam intersection angle, γ:

(1) $$E_{collision}=\frac{1}{2}\mu v_{r}^{2}=\frac{1}{2}\mu \left(v_{D2O}^{2}+v_{H2}^{2}-2v_{D2O}^{2}v_{H2}^{2}\cos\gamma \right)$$

where *v*_D2O_ and *v*_H2_ are the velocities of the D_2_O and H_2_ beams, *µ* is the reduced mass of the system D_2_O-H_2_, and *v_r_* is their relative velocity. The state-to-state ICS is measured by resonance-enhanced multiphoton ionization (REMPI) coupled with time-of-flight mass spectrometry (TOF-MS), using tunable UV/VUV lasers (see the Methods section for more details). We describe the characteristics of each beam, which has an impact on the experimental results before presenting the experimental ICSs.

The D_2_O or *normal*-H_2_ beams are generated using Even–Lavie valves. The measured characteristics of the gas pulses, velocity, pulse duration, velocity spread, and angle spread (see Section 4) are reported in Table 1.

#### 2.1.1. D_2_O Beam

**Figure 3:**
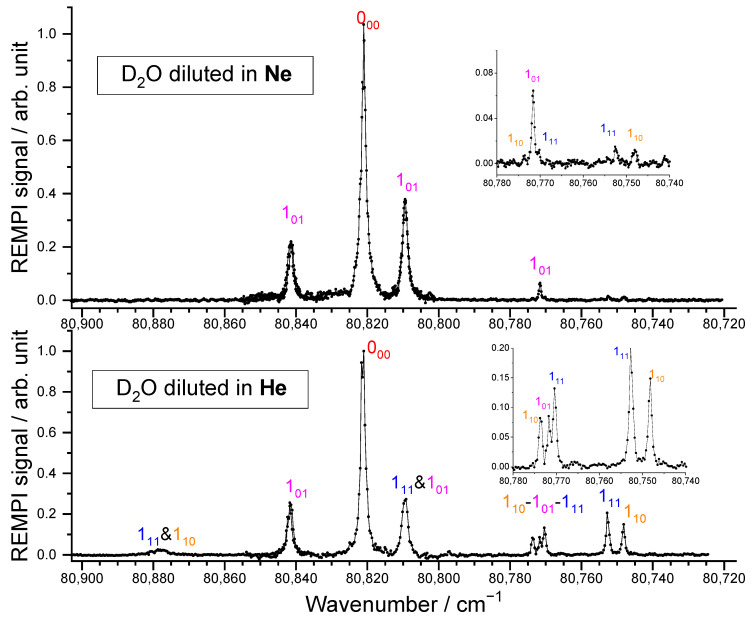
A (2 + 1) REMPI spectrum of the C^1^B_1_, v′ = 0 ← X^1^A_1_, v = 0 transition of D_2_O in the supersonic beam when Ne (top) or He (bottom) is used as a carrier gas. The inset is the enlargement of a part of the spectrum. The labels indicate the rotational levels, *j_kakc_*, from which the transitions originate. See Yang et al. [25] for more details.

Two carrier gases were used for the D_2_O beam, namely He and Ne, leading to beams with different characteristics (see Table 1). From the REMPI spectra reported in Figure 3, the D_2_O rotational distribution was determined using the spectroscopic data of the C^1^B_1_, v′ = 0 ← X^1^A_1_, v = 0 transitions, from Yang et al. [25] provided in PGopher software [26]. In our previous study on D_2_O + *para*-H_2_ [8], a rotational temperature of 12 K was determined for the D_2_O beam seeded in He, with a spin temperature of 320 K, corresponding to the reservoir temperature. The rotational populations were thus 0.55, 0.19, 0.15, 0.05, and 0.04, for the 0_00_, 1_01_, 1_11_, 1_10_, and 2_02_ levels, respectively. Further investigation of the contributions of the different rotational levels on the ICSs for the transitions to the D_2_O 2_02_ rotational level allowed us to refine the rotational population ratio of D_2_O (0_00_:1_11_) and (1_01_:1_10_) to 0.81:0.19 and 0.73:0.27, respectively. New experimental inelastic collision studies were performed with this beam of D_2_O diluted in He and supplemented with D_2_O seeded in Ne. For the latter beam, the REMPI spectrum is given in Figure 3. Obviously, only the lowest rotational levels of *ortho*-D_2_O and *para*-D_2_O are populated: the inset is the enlargement of the spectrum range where the first excited rotational levels may be seen and were estimated to have a population of less than 2%. As these signals are almost within the noise (see the inset of the D_2_O spectrum) even if the transitions are favored for the excited states, only the ground rotational levels will significantly contribute to the inelastic collisions.

Indeed, rotational cooling is more efficient when water molecules are seeded in Ne rather than in He [25]. However, even if the cooling is rapid, hence avoiding condensation, dimers may be formed, i.e., a weekly bound van der Waals structure, which presents shallow minima of ~34 and 7 cm^−1^ for D_2_O–Ne [27] and D_2_O–He clusters [28], respectively. Usually, a large cluster binding energy enhances the formation of clusters, which release their condensation energy into the beam, thence limiting translational cooling. However, the characteristics of the beams are equivalent with or without D_2_O. Moreover, no signal at higher *m*/*z* than those of D_2_O^+^ was detected. In conclusion, there is no evidence of significant amounts of water clusters in the beam.

As carrier gas mass changes, the beam velocity changes, too (see Table 1). The terminal velocity *v* of a supersonic beam generated by an adiabatic and isentropic expansion of an ideal gas through a nozzle, from a reservoir at a temperature *T_0_*, is defined by:

(2) $$\frac{1}{2}mv^{2}=\int_{T}^{T_{0}}C_{p}dT,$$

where the temperature of the beam is *T* << *T_0_* (substantial cooling in the expansion), and *C_p_* is the average molar heat capacity (for an atom, *C_p_* = 5/2 *R*, where *R* stands for the perfect gas constant). This equation implies a velocity ratio of √(20/4) = 2.2 for the two carrier gas, which is experimentally observed (see Table 1, where 1858/853 = 2.2).

#### 2.1.2. Normal-H_2_ Beam

**Figure 4:**
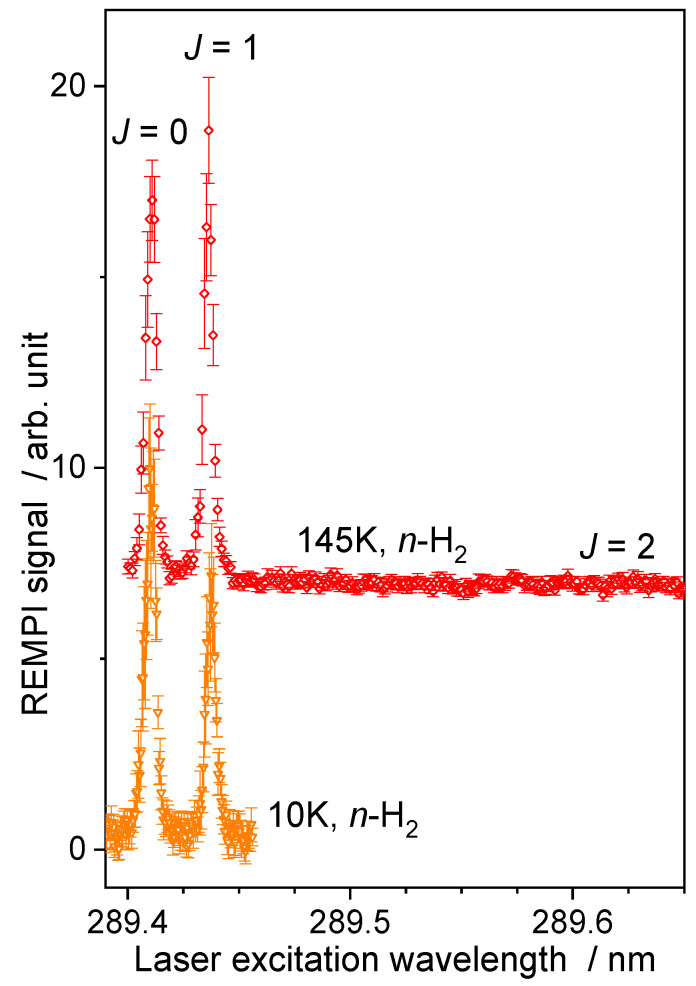
The (3 + 1) REMPI spectra [29] of normal-H_2_ beams. Temperature (145 K or 10 K) is the set point of the cold head. Vertical error bars correspond to statistical uncertainties at 95% of the confidence interval.

As the best collisional energy resolution is obtained when the two beams have almost the same velocity (Figure 2), for the H_2_ beam, a cryogenically cooled valve was used with He closed-cycle cryostats (Equation (2)). The temperature of the cold head was reduced at its minimum, which corresponds to a setpoint of 10 K to reach the lowest hydrogen beam velocity, which is almost equal to the neon beam velocity (Table 1). In the case of water diluted in He, a setpoint at 145 K was used. However, even if the temperature in the reservoir is decreased to a setpoint of 145 K or 10 K, there is no *ortho*-to-*para* conversion of the hydrogen. *Normal*-H_2_ beam remains a mixture of *para*- and *ortho*-H_2_ with the room temperature ratio, i.e., 3:1. The H_2_ beam was probed (Figure 4) when recording (C^1^Π_u_; *v* = 2 ← X^1^Σ^+^_g_; *v* = 0) R(0) and R(1) transitions near 96.47 nm, with a (3 + 1) REMPI scheme at 289.4 nm [29]. No evidence of R(2) signal was found for the beam with the highest reservoir temperature: a *normal*-H_2_ beam is composed of 25% of H_2_(*j_H2_* = 0) and 75% of H_2_(*j_H2_* = 1).

#### 2.1.3. Integral Cross-Sections

Three rotational levels of D_2_O were probed: 1_11_, 2_02_ for *ortho*-D_2_O, and 1_10_ for *para*-D_2_O. The REMPI intensities were recorded versus the angle relative to the beam axes. Collision energies were derived from Equation (1). The cross-section was obtained from the REMPI signal intensity, divided by the relative velocity of D_2_O and H_2_ and the interaction time of the two beams (see the Methods section for more details).

**Figure 5:**
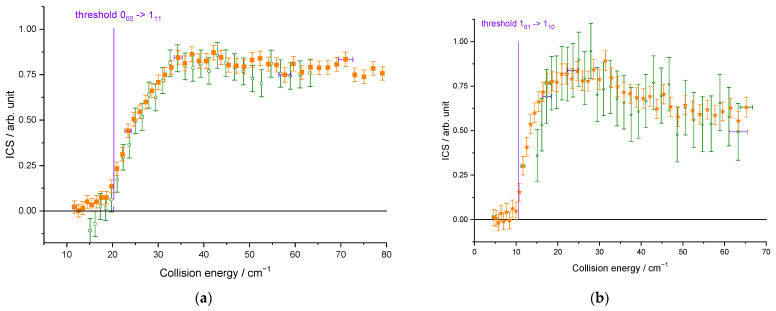
Experimental cross-sections for different conditions: in orange and in green, for D_2_O seeded in Ne and in He, respectively: (**a**) for the D_2_O(0_00_) → D_2_O(1_11_) transition; (**b**) for the D_2_O(1_01_) → D_2_O(1_10_) transition. Negative values for the cross-sections mean that the rotational population probed decreases: loss of the population on the 1_10_ level by transition to the 1_01_ or 2_12_ levels and loss of the 1_11_ population by transition to the 0_00_ or 2_02_ levels. Vertical error bars correspond to statistical uncertainties at 95% of the confidence interval.

**Figure 6:**
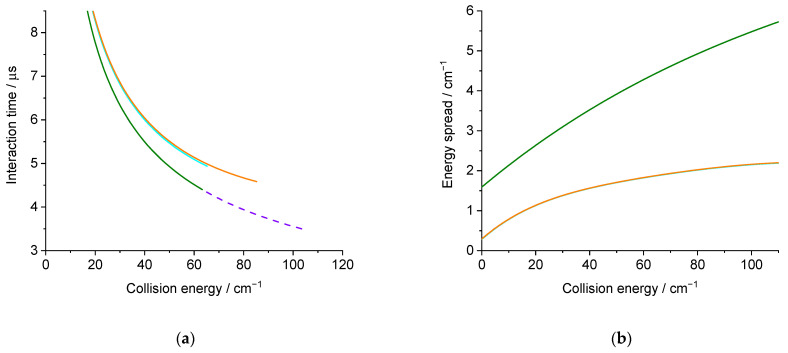
(**a**) Interaction time for an isotropic distribution; (**b**) collision energy spread calculated for the experimental conditions: in orange (green) solid lines, D_2_O seeded in Ne(He) for the D_2_O(0_00_) → D_2_O(1_11_) or D_2_O(1_01_) → D_2_O(1_10_) transition, and in purple dashed line, D_2_O seeded in He for the D_2_O(0_00_) → D_2_O(2_02_) transition.

The beam characteristics are given in Table 1. In Figure 5, whatever the seeded gas was for the water beam and thus the temperature of the hydrogen beam, the cross-sections are well reproduced: the thresholds are at the correct energies (20.3 and 10.6 cm^−1^ for the 0_00_–1_11_ and 1_01_–1_10_ excitation, respectively), and we can observe some resonances at the same energies. There is thus no evidence of any clusters in the beams, as the binding energies of He–D_2_O and Ne–D_2_O differ by 30 cm^−1^, and the D_2_O + H_2_ resonances are observed at the same collision energies for the two water carrier gases. However, compared to the ICSs measured when D_2_O is seeded in Ne, they are broadened and slightly less marked in the case of D_2_O seeded in He. In fact, the collision energy spread is larger (Figure 6), resulting in a spread-out of the resonance features over a more extended collision energy range. Moreover, the signal starts from a negative value in Figure 5a: the 1_11_ rotational population starts decreasing when H_2_ collides with D_2_O. Indeed, in the D_2_O:He beam, the first two rotational levels of *ortho*-D_2_O and *para*-D_2_O are populated, leading to the de-excitation of the 1_11_ excited state until the collision energy is large enough to promote the excitation from the ground state (0_00_ → 1_11_). When the 1_10_ excited rotational level is probed (Figure 5b), the lowest collision energy, which corresponds to our lowest angle between the two beams, is above the threshold (1_01_ → 1_10_), not allowing the observation of the de-excitation. In the case of D_2_O:Ne, only the ground rotational levels are populated (see Figure 3). Thus, in Figure 5, there is only one contribution from the ground rotational level to the probed excited one: the 0_00_ → 1_11_ and 1_01_ → 1_10_ transitions in Figure 5a,b, respectively.

Each experimental ICS datum corresponds to an average over a Gaussian distribution of collision energies: the half width at 1/e height, called collision energy spread, is given in Figure 6b. More details are given in the Methods section. This collision energy spread originates from the velocities and angular spread (Table 1). It will also affect the average relative velocity and the interaction time, which is given in Figure 6a. As the deconvolution of the experimental points is not possible, the theoretical cross-sections are convoluted with the collision energy spread to allow for comparison with the experimental data. Moreover, as previously explained, the rotational population distribution is also taken into account.

### 2.2. Theoretical Results

Inelastic scattering calculations were performed in a similar way to Bergeat et al. [8], and a summary can be found in the Methods section. The theoretical cross-sections for three particular rotational excitations from the ortho-D_2_O ground state by collisions with H_2_ are plotted in Figure 2 and Figure 3.

Calculations were performed for H_2_ in its ground *para* (*j_H2_* = 0) and *ortho* (*j_H2_* = 1) states, and cross-sections for *normal*-H_2_ were obtained for an *ortho*-to-*para* ratio fixed at 3. The rotational basis set for D_2_O included angular momenta up to *j_D2O_* = 6, corresponding to the lowest 25 *para*- and lowest 25 *ortho*-D_2_O levels. The lowest two levels were included in the basis set for *para*-H_2_ (*j_H2_* = 0, 2) and *ortho*-H_2_ (*j_H2_* = 1, 3). Enlarging the D_2_O or H_2_ basis sets was found to be negligible at the investigated energies, with cross-sections changing by less than one percent. We note, however, that because of the potential well (*D_e_* = 238 cm^−1^) and anisotropy of the D_2_O–H_2_ PES, the inclusion of closed-channels was found to be crucial, even for *ortho*-H_2_.

**Figure 7:**
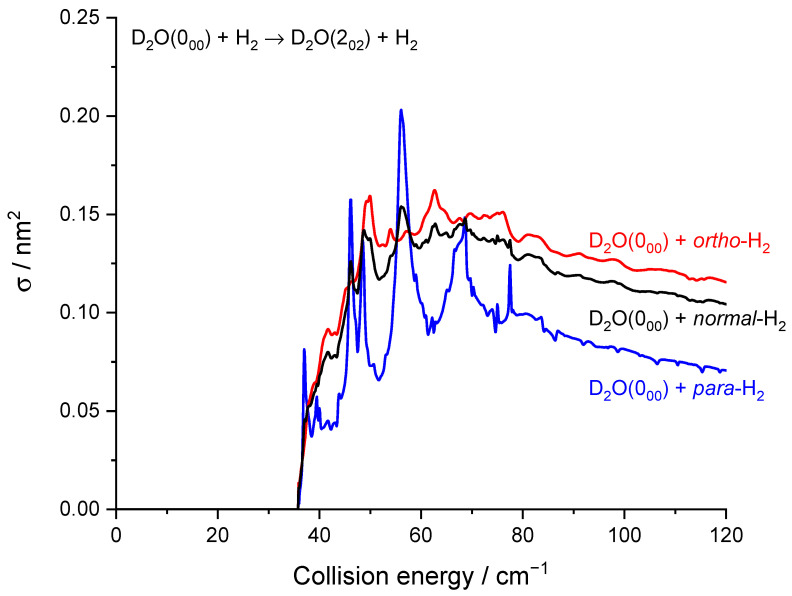
Rotational excitation cross-sections for the D_2_O 0_00_–2_02_ transition in blue *para*-H_2_(*j_H2_* = 0), in red *ortho*-H_2_(*j_H2_* = 1), and in black *normal*-H_2_(*j_H2_* = 0 and 1).

**Figure 8:**
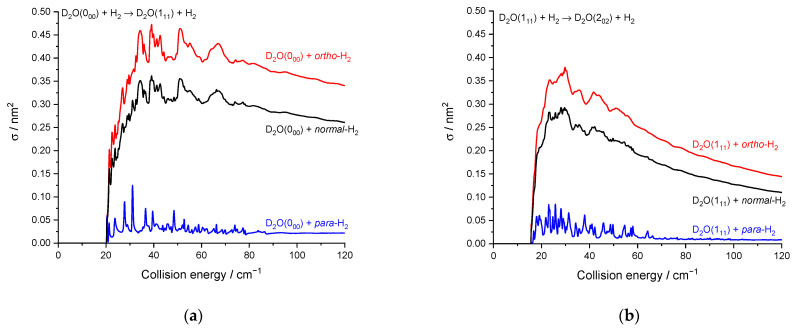
Rotational excitation cross-sections in blue D_2_O + *para*-H_2_(*j_H2_* = 0), in red D_2_O + *ortho*-H_2_(*j_H2_* = 1), and in black D_2_O + *normal*-H_2_(*j_H2_* = 0 and 1): (**a**) for the 0_00_–1_11_ transition; (**b**) for the 1_11_–2_02_ transition.

As previously noted for the H_2_O–H_2_ system [17] and also for other molecule-H_2_ systems [30], resonances are more conspicuous for *para*-H_2_ (*j_H2_* = 0) than for *ortho*-H_2_ (*j_H2_* = 1). This is particularly striking here for the 0_00_ → 2_02_ transition (Figure 7) where resonances have magnitudes up to twice that of the background cross-section for *para*-H_2_ (*j_H2_* = 0) while for *ortho*-H_2_ (*j_H2_* = 1) are much less intense. This can be explained by the non-vanishing quadrupole of *ortho*-H_2_ (*j_H2_* = 1) and the corresponding stronger binding with *ortho*-H_2_ (*_jH2_* = 1) than with *para*-H_2_ (*j_H2_* = 0). This was observed in the H_2_O–H_2_ complex where the dissociation energy and number of bound-states are indeed significantly larger for *ortho*-H_2_ (*_jH2_* = 1) monomers than *para*-H_2_ (*j_H2_* = 0) monomers [31]. Overlapping and broadened resonances are thus expected to be more numerous in D_2_O-*ortho*-H_2_ (*j_H2_* = 1) with respect to D_2_O-*para*-H_2_ (*j_H2_* = 0). In addition, it is observed that the background cross-sections for *para*-H_2_ (*j_H2_* = 0) and *ortho*-H_2_ (*j_H2_* = 1) have similar magnitudes for the transition 0_00_ → 2_02_ while *ortho*-H_2_ (*j_H2_* = 1) cross-sections are much larger than *para*-H_2_ (*j_H2_* = 0) cross-sections for other transitions, e.g., 0_00_ → 1_11_ and 1_11_ → 2_02_ (Figure 8). This is clearly due to the dipole–quadrupole interaction, which dominates in D_2_O dipolar transitions (i.e.*,* those with Δ*j_D2O_* = 0, ±1, ∆*k_a_* = ±1, ∆*k_c_* = ±1) while D_2_O quadrupolar transitions (∆*j_D2O_* = ±2) are much less sensitive to the quadrupole of H_2_.

## 3. Discussion

**Figure 9:**
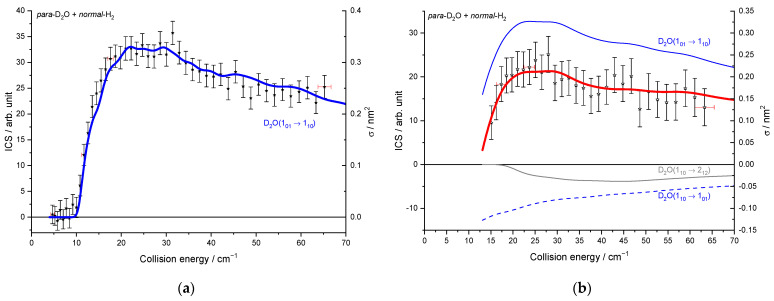
Experimental and theoretical cross-sections for the D_2_O + *normal*-H_2_ → D_2_O(1_10_) + *normal*-H_2_ inelastic collisions. Black squares are experimental ICSs for the 1_10_ rotational level, with error bars corresponding to statistical uncertainties at 95% of the confidence interval, in the conditions of (Table 1): (**a**) D_2_O seeded in Ne and *normal*-H_2_ at 10 K (**b**) D_2_O seeded in He and *normal*-H_2_ at 145 K. Negative values for the cross-sections mean that the 1_10_ rotational population probed decreases. In red solid line, the total theory contributions are convoluted to experimental resolution, with partial contributions in blue, the 1_01_ → 1_10_ transition, in blue dotted line, the loss is due to the 1_10_ → 1_01_ transition, and in gray, the loss is due to the 1_10_ → 2_12_ transition, assuming the initial rotational population given in Table 2.

**Figure 10:**
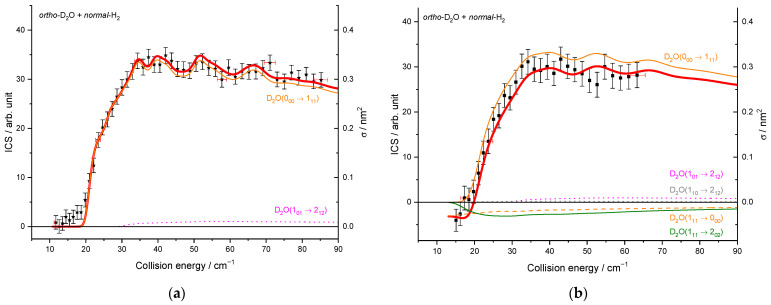
Experimental and theoretical cross-sections for the D_2_O + *normal*-H_2_ → D_2_O(1_11_) + *normal*-H_2_ inelastic collisions. Black squares are experimental ICSs for the 1_11_ rotational level, with error bars corresponding to statistical uncertainties at 95% of the confidence interval, in the conditions (Table 1) of: (**a**) D_2_O seeded in Ne and *normal*-H_2_ at 10 K (**b**) D_2_O seeded in He and *normal*-H_2_ at 145 K. Negative values for the cross-sections mean that the 1_11_ rotational population probed decreases. In red solid line, the total theory contributions are convoluted to experimental resolution, with partial contributions of the 0_00_ → 1_11_ transition (in orange solid line), the 1_01_ → 2_12_ transition (in magenta dotted line), 1_10_ → 2_12_ transition (in gray), the loss due to the 1_11_ → 2_02_ transition (in olive), and the loss due to the 1_11_ → 2_12_ transition (in orange dotted line), assuming the initial rotational population and a partial contribution from the 2_12_ state, when probing the 1_11_ state, given in Table 2.

**Figure 11:**
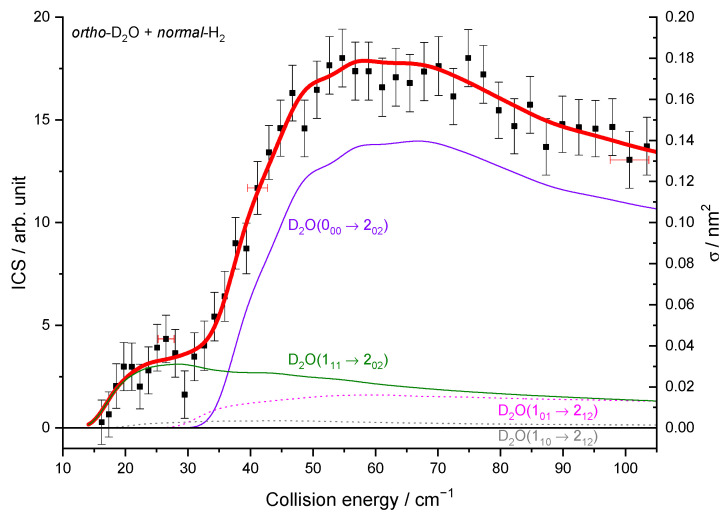
Experimental and theoretical cross-sections for the D_2_O + *normal*-H_2_ → D_2_O(2_02_) + *normal*-H_2_ inelastic collisions in the conditions (Table 1) of D_2_O seeded in He and *normal*-H_2_ at 145 K. Black squares are experimental ICSs for the 2_02_ rotational level, with error bars corresponding to statistical uncertainties at 95% of the confidence interval. In red solid line, the total theory contributions are convoluted to experimental resolution, with partial contributions in violet solid line, the 0_00_ → 2_02_ transition, in olive, the 1_11_ → 2_02_ transition, in magenta dotted line, the 1_01_ → 2_12_ transition, and in gray 1_10_ → 2_12_ transition, assuming the initial rotational population and a partial contribution from the 2_12_ state, when probing the 2_02_ state, given in Table 2.

To compare the experimental results with the theoretical cross-sections, the calculated ICSs were convoluted with the experimental collision energy spread, and the different contributions of the rotational populations were added or subtracted with the appropriated weights (Table 2), as explained in the Methods section. To begin, the *para* to *ortho* ratios were kept constant at their values at 320 K as the *ortho*–*para* conversion rates are extremely slow without any magnetic catalyst: 2:1 for D_2_O and 3:1 for H_2_ (see Table 2). In Figure 9a, only one state-to-state contribution is observed, the D_2_O(1_01_) + *normal*-H_2_ → D_2_O(1_10_) + *normal*-H_2_. This is because only the lowest *para*- and *ortho*-D_2_O rotational levels are significantly populated when seeded in Ne. The overall excitation function, i.e., the evolution of the ICS versus the collision energy, is in good agreement with the convoluted theoretical calculations. In the case of the excitation transition from D_2_O(0_00_) to D_2_O(1_11_) shown in Figure 10a, small undulations due to resonances are observed in perfect agreement with the quantum mechanical calculations. However, we should underline that a small contribution of 0.12 on the 1_01_ to 2_12_ transition was added to take into account the overlap of the REMPI peak from the probed 1_11_ rotational level of D_2_O with those from the 2_12_ level. Then, the REMPI intensities for the D_2_O(1_10_) were recorded when heavy water was seeded in He (Figure 9b). Due to higher beam velocities, the minimum collision energy probed is above threshold. Furthermore, the signal is small as some population in the excited state is lost by de-excitation to the ground state and excitation to the next upper state. No real structure was observed. The 1_10_ to 1_01_ population ratio of the water beam in He was deduced from this excitation function and used for the other fits, despite the uncertainty. The 1_11_ to 0_00_ population ratio was then fitted from the 2_02_ experimental ICSs (Figure 11), using the overlap value of the REMPI peaks from 2_12_ and 2_02_ determined previously [8]. All these population ratios and REMPI peak overlaps summarized in Table 2 were also used to analyze the ICSs of the D_2_O(1_11_) transitions when water is seeded in He. The very good agreement between theory and experiment in Figure 10b, confirmed the accuracy of our experimental treatment and the theoretical calculations.

The resonances observed for the 0_00–_2_02_ rotational inelastic cross-sections occur because of quasi-bound states become transitorily accessible during the collision. They were characterized by analyzing partial cross-sections differing by their total angular momentum *J* [8]. The total cross-section (summed over all partial waves *J*) was found to show, on resonance, large contributions from specific orbital angular momentum *l*, the rotational states of H_2_, and D_2_O being *j_D2O_* = 0 and *j_H2_* = 0. An analysis of the resonances was put forward in the preceding paper [8].

The experiments conducted with *normal*-H_2_ at a given collision energy can only observe the summation of all partial wave contributions weighted by the (2*J* + 1) degeneracy factor in the ICSs. The contributions of many overlapping partial waves corresponding to the energetically allowed *J*-values tend to average the individual resonance amplitudes, making them interfere and average out. Unfortunately, for the processes with *para*-H_2_, as explained previously (see Figure 2 and Figure 3), the theoretical calculations predict too small transition probabilities to be experimentally probed at the collision energies close to the thresholds, except for the 0_00_ to 2_02_ rotational excitation of D_2_O [8].

In summary, we were able to observe some resonances in the experimental excitation functions, and the agreement between experiment and theory is excellent in all cases presented herein, for the collision energy range 10–100 cm^−1^.

**Figure 12:**
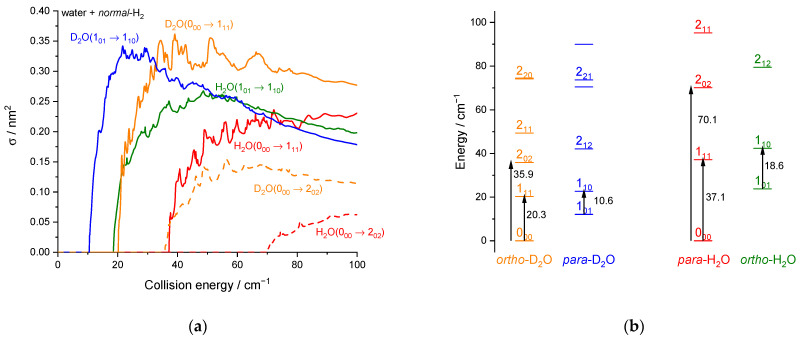
D_2_O or H_2_O + *normal*-H_2_ rotational inelastic collisions: (**a**) Theoretical cross-sections of the D_2_O 0_00_ → 1_11_ transition in orange solid line, the D_2_O 0_00_ → 2_02_ transition in orange dashed line, the D_2_O 1_01_ → 1_10_ transition in blue line, the H_2_O 0_00_ → 1_11_ transition in red solid line, the H_2_O 0_00_ → 2_02_ transition in red dashed line, and the 1_01_ → 1_10_ transition in olive line. (**b**) Rotational energy diagram up to *j_1_* = 2 with the four transitions shown in panel (a) [7].

While the reduced mass of D_2_O + H_2_ (1.8313 u) is only slightly different from that of H_2_O + H_2_ (1.8128 u), the water deuteration can significantly affect inelastic collisional rates: the H/D isotopic substitution results in a difference in the reduced mass, rotational constants, zero-point energies and energies of the quasi-bound states supported by the potential. Additionally, the center of mass is slightly moved. Consequently, the intensities and positions of the resonant peaks will vary. In Figure 12, we have reported the ICSs calculated for the first rotational excitation of D_2_O and H_2_O by collisions with *normal*-H_2_. To ease the comparison, the rotational energy diagram of H_2_O and D_2_O was added. Note that *ortho* and *para* systems are inverted due to the nuclear spins (deuteron versus proton).

We can observe in Figure 12 that the first rotational transitions 0_00_ → 1_11_ and 1_01_ → 1_10_ for H_2_O + H_2_ and D_2_O + H_2_ inelastic collisions. Just after the threshold rise and before the decrease, the excitation ICSs for H_2_O tend to be smaller than those for D_2_O by typically 30% (in qualitative accordance with the energy gap law) while the propensity rules (discussed in the theoretical results section in terms of the dipolar transitions in the C_2v_ symmetry) are equivalent for these *b*-type transitions (∆*k_a_* = ±1, ∆*k_c_* = ±1). At larger collision energies, the differences between D_2_O and H_2_O tend to be smaller. Thus, for these two dipolar transitions, the differences between D_2_O and H_2_O are not substantial and mainly reflect the kinematics effects (mass and velocities) due to the change in rotational constants and the corresponding different rotational thresholds. For dipole-forbidden transitions, however, much larger differences are observed, as observed in Figure 12a for the transition 0_00_ → 2_02_ where the cross-section peak for D_2_O exceeds the cross-section plateau for H_2_O by a factor of ~ 2.5. As explained by Scribano et al. [20], this is due to the non-dipolar interaction terms in the PES, which are more affected by the isotopic substitution than the dipolar terms. As a result, the H/D substitution results both in kinematics and PES effect whose relative importance depends on the type of transition.

## 4. Methods

### 4.1. Theoretical Calculations

As in Bergeat et al. [8], inelastic scattering calculations were performed at the close-coupling level with the MOLSCAT program [32] using a rigid-rotor D_2_O–H_2_ potential energy surface (PES) adapted from the full-dimensional H_2_O–H_2_ PES of Valiron et al. [22]. The shift of center of mass and change of internal geometries (zero-point vibrational effects) were both taken into account. Full details about the geometrical transformation from H_2_O–H_2_ to D_2_O–H_2_ can be found in [20], but it should be noted that we found a small error in the implementation of Scribano et al. [20] so that in practice a new rigid-rotor D_2_O–H_2_ PES was used in [8] and also herein.

D_2_O is an asymmetric top, and the rotational constants were taken as *A* = 15.41998, *B* = 7.27299, and *C* = 4.84529 cm^−1^. Centrifugal correction terms were unnecessary for the low-lying levels examined herein. For H_2_, the rotational constant is *B_0_* = 59.322 cm^−1^, as in Bergeat et al. [8]. The reduced mass of the D_2_O–H_2_ system is 1.8313 u. The coupled differential scattering equations were solved using the hybrid modified log-derivative Airy propagator for total energies up to 300 cm^−1^.

Convergence was carefully checked with respect to the propagator step size. Finally, we used a fine energy grid of 0.25 cm^−1^, and the cross-sections were interpolated with a cubic spline in order to convolve them with the experimental collision energy spread (≳ 1 cm^−1^).

### 4.2. Crossed Molecular Beams

#### 4.2.1. Experimental Procedure

We performed our experiments with a crossed-beam apparatus (Figure 2), with a fixed water beam and a rotatable H_2_ beam.

The gas manifold comprises a 0.5 L steel tank of high purity liquid deuterated water into which pure neon or helium is added at 12 bar. The tank is held at 40 °C, resulting in a 0.8578 kPa D_2_O vapor pressure [33] and 0.07% D_2_O/Ne or He gas mixture. This gas mixture is then introduced into the stagnation region of a pulsed Even–Lavie valve (100-µm aperture, 150-µm conical nozzle, ~10 µs pulse-time, 10 Hz repetition rate) maintained at 320 K to avoid any condensation of water before the supersonic expansion. A turbomolecular pump keeps an average chamber pressure of ∼10^−6^ mbar under full pulsed valve operation. The resulting D_2_O beam seeded in Ne or He travels ∼95 mm through the source chamber, separated to the main chamber by a 3-mm inner diameter skimmer. Then, the supersonic molecular beam flies ~22 cm to the interaction region (with a background pressure < 10^−6^ mbar), where the second supersonic beam crosses, in perfect synchronization.

*Normal*-H_2_ beam pulses were obtained using cryogenically cooled Even–Lavie valve (pulse-time of 7.3 and 5.8 µs for a valve setpoint at 145 or 10 K, respectively) with 12.5–13.5 bar of net hydrogen in the reservoir. The pulsed valve chamber is maintained at ∼10^−6^ mbar by a turbomolecular pump and is separated to the main chamber by a 2-mm skimmer 33 mm after the nozzle. This H_2_ source chamber may rotate from 90° to 13° allowing the beam intersection angle to be varied continuously. The distance from the skimmer to the crossing point is ~6 cm and the time delay between the two pulsed beams was adjusted to ensure a perfect overlap of both beams at the crossing point [24]. To minimize background contributions from any rotationally excited molecules in the water beam or the main chamber, the H_2_ beam is triggered at 5 Hz, with signals averaged in alternating pulse mode and recorded as two intensities: signal and background.

Detection of D_2_O rotational levels in its (X^1^A_1_, *v* = 0) ground vibronic state is achieved in the beam-crossing region by a laser beam perpendicular to the scattering plane. D_2_O is detected by REMPI with a 2 + 1 scheme in the UV: two-photon resonant and one-photon ionization at ~247 nm via the (C^1^B_1_, *v*’ = 0) Rydberg state. The laser system consisted of a dye laser (Coumarin 500) pumped by the third harmonic at 355 nm of an Nd:YAG laser. UV laser pulse energies of 2.5–4.5 mJ per pulse at repetition rate of 10 Hz were generated by doubling the visible laser output in a beta barium borate (BBO) crystal. A small part of the visible dye laser was sent to a wavemeter to control the D_2_O rotational state probed, and a photodiode was monitoring the UV laser to ensure that the energy remained constant during scanning. A two-stage time-of-flight mass spectrometer (TOF MS), which has specially cut plates to allow the beams to pass through, is positioned in the beam scattering plane and tilted by 135° to the water supersonic beam. A multichannel plate detector is used for ion detection. The signal is pre-amplified, gated, and integrated using a boxcar over the time-of-flight corresponding to the D_2_O mass and recorded to a computer.

To measure velocity distribution, the density temporal profiles of D_2_O by REMPI-MS detection at the crossing point and of Ne or He by a fast ionization gauge (FIG) at *d* = 393.3 mm downstream were recorded and fitted to Gaussian profiles. The detailed procedure was used to extract the density pulse profile, and the velocity distribution was detailed previously [24,34]. Briefly, the Gaussian functions with peak positions *t_0_* and *t_1_* and widths *γ_0_* and *γ_1_* yield the peak velocities *v* = *d*/(*t_1_* − *t_0_*), and the velocity spreads from the pulse broadening, taking into account the response time of the fast-ionization gauge. The results are reported in Table 1. To measure the angular divergence of the beams, the TOF-MS was replaced by a second FIG inserted perpendicular to the beam axis. The two density profiles obtained by the two FIGs separated by *d* = 393.3 mm were measured at different positions of the FIGS perpendicularly to the beam axis: Gaussian fits gave the angular spread reported in Table 1 and the exact beam axis locations. It should be noticed that the beam velocity characterizations obtained by the two FIGs or by the association of the REMPI signal with the far FIG gave the same results.

ICSs were obtained from the REMPI signal intensities of the rotationally excited water molecules, D_2_O(*j_kakc_*). The intensities, signal, and background were acquired for each individual laser shot and intersection angle. The points associated with too small UV laser intensities were rejected to stay in the linear regime whereby the REMPI intensity only depends on the water density. After subtraction of the background mean for each angle, each signal intensity was normalized by the average value over the whole angle scan, allowing for accumulation of several short-duration scans measured in 2 or 3 days. The conditions for each result set presented are summarized in Table 3. All the plotted vertical error bars on the experimental ICSs represent the combination of statistical fluctuations at a 95% confidence interval of the signal and background data. The plotted error bars on energy are estimated from velocity and crossing angle uncertainties: it represents the uncertainty of the peak collision energy value, not the collision energy spread that is reported in Figure 6b.

The experimental ICSs were obtained from the averaged REMPI signal intensities *I*, the relative velocity *v_r_* of the D_2_O and H_2_ beams, and the mean interaction time ∆*t* between the two beam pulses, as σ = *I*/(*v_r_* ∆*t*). This interaction time, given in Figure 6a, takes full account of the density-to-flux transformation under our working conditions, when modelling pulsed beam densities with spatial and temporal Gaussian distribution functions.

**Figure 13:**
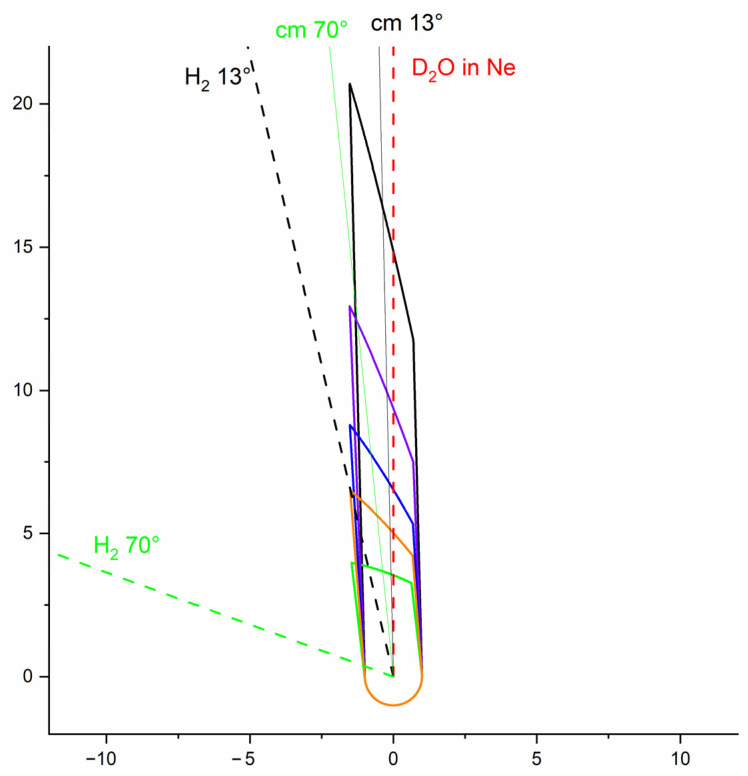
The interaction volumes taken into account in the density-to-flux transformation for different crossing angles relatively to the water beam: in black 13°, in violet 21°, in blue 31°, in orange 42°, and in green 70°. The red dashed line is the D_2_O:Ne beam axis. The black and green dashed lines are the H_2_ beam axis, and the black and green solid lines are the center-of-mass velocity vectors, for a crossing angle of 13° and 70°, respectively.

Indeed, the beam crossing region where collisions contribute to the signal is delimited by the waist of the laser beam steered perpendicularly to the beam scattering plane and also by the beam overlap region even far upstream from the crossing point (see Figure 13). In fact, we consider the locus of points (*x*, *y*) for which the product of pulse beam densities *n*_H2_(*x*, *y*, *t*) x *n*_D2O_(*x*, *y*, *t*) = 1/e. As at low collision energies (or angles), which correspond to near-threshold collisions, the excited water molecules have almost no recoil energies (or velocities); the scattered D_2_O are therefore travelling in phase with the center-of-mass (cm) velocity vector. For each collision energy, there is only one definite value for the velocity of the recoiling product, the rotationally excited D_2_O, in the center-of-mass frame (Figure 2b). Under all our experimental conditions, the scattered D_2_O product recoils with a velocity in the cm frame, which remains rather low (in the most unfavorable case: 82 m s^−1^ for a *v_cm_* = 809 m s^−1^); its velocity in the laboratory frame thus remains very close to the cm velocity. This induces not only an angular spread and a collision energy spread (Figure 6b) but also a highest value of the mean collision energy at a specific angle relative to the beam axes, *γ* due to a shift of the mean value of crossing angle [34]. In the ∆*t* calculations, the angular distribution of scattered species was also taken into account. However, in our previous study on inelastic collisions between D_2_O and *para*-H_2_ [8], the differential cross-sections were also calculated, and it was shown that the collisions were backward, forward, backward-forward, or istropic: the dynamics were varying with the collision energy. As the angular distribution (forward or isotropic) was found to have an almost negligible impact on the time-interaction values, we have simplified the analysis, considering an isotropic distribution of the products at all collision energies for all the rotationally excited D_2_O products.

#### 4.2.2. Data Treatment for Comparisons

The total inelastic collision cross-section *σ_i_*_→*f*_ is related to the overall probability that an inelastic transition will take place from the rotational state |*i*> of D_2_O to the state |*f*> by collisions with *normal*-H_2_. The bimolecular rate will induce a transition |*i*> → |*f*> in D_2_O is:

(3) $$\frac{dn_{f}}{dt}=n_{H2}n_{i}k_{i\to f}\left(v_{r}\right)=n_{H2}n_{D2O}p\left(i\right)v_{r}\sigma_{i\to f}\left(v_{r}\right)$$

where *k_i_*_→_*_f_ (v_r_)* is the rate constant at relative velocity *v_r_*_,_ *n_H2_* the number density of the H_2_ collision partner, *n_D2O_* the D_2_O number density, and *p(i)* the population of D_2_O in the rotational state |*i*>.

The rate law for the inelastic collisions between D_2_O and H_2_ of a particular probed rotational level |*f*> density (*n_f_*) is thus:

(4) $$\begin{array}{l} \frac{dn_{f}}{dt}=n_{H2}n_{D2O}\sum_{i\neq f}p\left(i\right)k_{i\to f}\left(v_{r}\right)-n_{H2}n_{D2O}p\left(f\right)\sum_{l\neq f}k_{f\to l}\left(v_{r}\right)\\ \;=n_{H2}n_{D2O}v_{r}\left(\sum_{i\neq f}p\left(i\right)\sigma_{i\to f}\left(v_{r}\right)-p\left(f\right)\sum_{l\neq f}\sigma_{f\to l}\left(v_{r}\right)\right) \end{array}$$

where the first term corresponds to filling up the state |*f*>, and the second term is related to the loss of population out of this initial level *f* level. *σ_i_*_→_*_f_* are the cross-sections from all the populated levels |*i*> to the |*f*> state when the relative velocity between D_2_O, and H_2_ is equal to *v_r_*, *p(i)* is the population ratio in the state |*i*>, and *σ_f_*_→_*_l_* the cross-sections of the |*f*> → |*l*> transitions if the probed level |*f*> is initially populated (*p(f)*). The REMPI intensity is proportional to the D_2_O density of the *f* rotational level probed, *n_f_*. However, as we do not know the exact densities of D_2_O and H_2_ in our beams, the experimental ICSs are given in arbitrary units. To compare the experimental ICSs with the theoretical ones, we have thus added or subtracted the calculated cross-sections weighted by the appropriate initial rotational population (see Table 2).

Moreover, at the wavelength of 247.34 or 247.67 nm chosen to probe the 2_02_ or 1_11_ rotational level of D_2_O, there is a small contribution from the 2_12_ level. Thus, the ICSs of the 1_01_ → 2_12_ and 1_10_ → 2_12_ were considered with weights taking into account the relative rotational populations and the relative strength of the REMPI transitions from the 2_02_ or 1_11_ and 2_12_ levels (see Table 2).

Each theoretical cross-section is calculated for one collision energy or relative velocity of D_2_O and H_2_ (Equation (1)), but the experimental values are resulting from the integration over collision energy spread. To compare theory and experiment, the calculated excitation functions were convoluted with our experimental energy spread (Figure 2), and the experimental data were multiplied by a unique scaling value.

## Figures and Tables

**Table 1:** Characteristics of the molecular beams used in the determination of the integral cross-sections.

Beam	Velocity ^1^ /m s^−1^	Velocity Spread ^1^ HWHM/m s^−1^	Pulse Duration ^2^ HWHM/µs	Angle Spread ^3^ HWHM/°
D_2_O in He	1858 ± 30	44	15.0	0.79
*normal*-H_2_ at 145 K	1959 ± 30	44	7.9	1.17
D_2_O in Ne	853 ± 24	14	19.3	0.71
*normal*-H_2_ at 10 K	947 ± 28	49	11.5	1.17

^1^ Beam velocity peak values and half width at half maximum (HWHM) of the velocity spread deduced from temporal profiles at the crossing point and 393.3 mm downstream. ^2^ Pulse duration (HWHM) at the crossing point. ^3^ Angular divergence (HWHM).

**Table 2:** Rotational population distributions in the D_2_O beam seeded in He or Ne and contribution to the main rotational level probed by another rotational level.

Contributions	D_2_O in He	D_2_O in Ne
*ortho*-D_2_O:*para*-D_2_O ^1^	2/3:1/3	2/3:1/3
0_00_:1_11_^2^	90:10	100:0
1_01_:1_10_^2^	83:17	100:0
2_12_ in 2_02_^3^	0.20	
2_12_ in 1_11_^3^	0.12	0.12

^1^ The *ortho*-to-*para* ratio of D_2_O remains at the room temperature equilibrium ratio of 2. ^2^ The relative population ratio of the two *ortho*- or *para*-D_2_O first rotational levels. ^3^ The relative contribution of the 2_12_ rotational level to the signal of the 2_02_ or 1_11_ level due to an overlap of the lines in the REMPI spectra (see the text for details).

**Table 3:** Each experimental ICSs of a specific rotationally excited water molecules, D_2_O(*j_kakc_*) corresponds to a number of laser shots, *n*, per angle, scanning the beam intersection angle in the range *γ_initial_*–*γ_final_* with −1° or −0.5° decrement.

D_2_O(*j_kakc_*)	Wavelength	*n* Laser Shots/Angle	Intersection Angles
2_02_	247.340 nm	2275	35°–13° (−0.5°)
1_11_ with D_2_O in Ne	247.670 nm	3585	70°–23°(−1°)
1_11_ with D_2_O in He	247.670 nm	3691	27°–12.5°(−0.5°)
1_10_ with D_2_O in Ne	247.684 nm	2482	60°–13°(−1°)
1_10_ with D_2_O in He	247.684 nm	2684	27°–12.5°(−0.5°)

## Data Availability

Data is contained within the article and may be obtained in another format on request from the authors.

## Sample Availability

Samples of the compounds are available from the authors.
